# Exploring Public Interest in Atrial Fibrillation and Its Treatment Measures: A Google Trends Analysis

**DOI:** 10.7759/cureus.83650

**Published:** 2025-05-07

**Authors:** Mukesh Ram Kumar Kommu, Haezel Ann Shibu, Elias Abboud, Manaswini Chowdary Kaka, Muhammad Usma Khalid, Kesha Pathak

**Affiliations:** 1 Cardiology, Rangaraya Medical College (Affiliated With Dr. NTR University of Health Sciences), Kakinada, IND; 2 Internal Medicine, Jawaharlal Institute of Postgraduate Medical Education and Research, Pondicherry, IND; 3 Internal Medicine/Surgery, Saint Joseph University of Beirut, Beirut, LBN; 4 Internal Medicine, American International Medical University, Gros Islet, LCA; 5 Internal Medicine, Services Hospital Lahore, Lahore, PAK; 6 Internal Medicine, Gujarat Adani Institute of Medical Sciences (GAIMS), Bhuj, IND

**Keywords:** amiodarone, artificial cardiac pacemaker, atrial fibrillation, flecainide, google trends, metoprolol, palpitations, tachycardia, united states

## Abstract

Introduction: Google Trends is a tool that analyzes the frequency of search terms over time, providing insights into public interest and behavior regarding various health topics. This research aims to utilize Google Trends to assess public interest in atrial fibrillation (AF) and inform healthcare strategies accordingly. The data provided by Google Trends is called relative search volume (RSV). This study evaluates changes in interest related to AF, including its medications and procedures, in the United States over 10 years.

Methodology: A cross-sectional original research study was conducted in July 2024 using the data obtained from Google Trends. The data collected were then exported to Microsoft Excel (Microsoft Corp., Redmond, WA, US) and analyzed using statistical methods.

Results: The RSV for all conditions showed an upward trend from 2014 to 2023. The RSV for “AF” was constant from 2014 to 2018, while “tachycardia” remained constant from 2014 to 2021. Searches for “AF” consistently exceeded those for “tachycardia” and “palpitations.” Interest in medications such as “metoprolol,” “diltiazem,” “flecainide,” “sotalol,” “amiodarone,” “dofetilide,” and “aspirin” also increased. Similarly, the RSV for procedures like “cardioversion”, “artificial cardiac pacemaker,” and “left atrial appendage occlusion” also rose over the past five years.

Conclusion: This study demonstrates a rising public interest in AF. Searches related to medications such as amiodarone, metoprolol, diltiazem, and flecainide, as well as procedures including cardioversion, artificial cardiac pacemaker, and left atrial appendage occlusion, have all shown increasing trends.

## Introduction

Atrial fibrillation (AF) is the most prevalent cardiac arrhythmia, characterized by irregular and often rapid heartbeats caused by abnormal electrical impulses in the atria. These impulses disrupt the heart’s natural rhythm, leading to irregular and accelerated contractions [1]. This disruption not only produces symptoms such as palpitations and dizziness but also significantly increases the risk of stroke, heart failure, and other cardiovascular complications, thereby contributing to substantial morbidity and reduced quality of life. A thorough understanding of AF's pathophysiology and its clinical impact is crucial for effective management and treatment.

Management of AF involves lifestyle modifications, medications, and procedural interventions. Key lifestyle changes involve maintaining a healthy weight, avoiding stimulants and alcohol, and quitting smoking [2]. Medications used in AF management encompass beta-blockers and calcium channel blockers for heart rate control; anticoagulants, such as warfarin and direct oral anticoagulants (DOACs) to reduce stroke risk; and antiarrhythmic drugs for rhythm control [2]. Recent advances include catheter ablation techniques, such as cryo-balloon ablation, and left atrial appendage closure devices to further prevent stroke [3]. Emerging therapies, such as pulsed field ablation, offer promise for improved efficacy and safety. Electrical cardioversion and pacemaker implantation remain viable options for managing persistent AF. Additionally, wearable devices support early detection and continuous monitoring, though they may also pose challenges such as false positives, potentially leading to unnecessary anxiety and interventions [4].

Google Trends is a tool that analyzes the frequency of search terms over time, providing insights into public interest and behavior regarding various health topics [5]. It aggregates and anonymizes search data, presenting results as a relative search volume (RSV), a measure of a term's popularity on a scale from 0 to 100. This approach allows researchers to identify trends in public interest without disclosing exact search volumes or user identities. Search trends may be influenced by factors such as media coverage, public events, and seasonal variations. Moreover, the data lacks demographic specificity and may not always accurately reflect actual healthcare utilization or clinical diagnoses. In this study, Google Trends is used to evaluate how public awareness and interest in AF and its treatment options have changed from 2014 to 2023. Understanding these trends can support the development of targeted public health campaigns, educational initiatives, and informed healthcare resource allocation to better address emerging patient concerns.

Aims and objectives

This study aims to analyze public interest in AF and its various treatment modalities in the United States over the past 10 years using Google Trends.

## Materials and methods

A cross-sectional original research study was conducted in July 2024. As no human participants were involved, the study was deemed exempt from Institutional Ethics Committee approval.

Google Trends is a freely accessible online analytics tool developed by Google (Google LLC, Mountain View, CA, USA) that enables the analysis of public interest in specific topics by generating graphical representations of search interest over time and across regions [6]. It offers potential utility in medical research by keeping healthcare professionals informed about evolving health-related interests. Google Trends uses RSV to quantify search data, representing the proportion of searches for a specific term relative to the total number of searches within a defined geographical area and time frame [7]. For this study, data were collected for the United States from January 1, 2014, to December 31, 2023, using the “All categories” and “Web search” filters. Disease-related terms were searched together using the following keywords: “Atrial Fibrillation”, “Tachycardia”, and “Palpitation”. However, treatment-related terms, including medications, procedures, and surgeries, were searched individually using the following keywords: “Digoxin”, “Metoprolol”, “Verapamil”, “Diltiazem”, “Procainamide”, “Disopyramide”, “Flecainide”, “Propafenone”, “Sotalol”, “Amiodarone”, “Dofetilide”, “Aspirin”, “Warfarin”, “Cardioversion”, “Pulmonary vein ablation”, “Artificial cardiac pacemaker”, “Left atrial appendage occlusion”, and “MAZE procedure”.

The collected data were then exported to Microsoft Excel (Microsoft Corporation, Redmond, WA, USA) for organization and further analyzed using R version 4.3.2. (R Core Team (2023), R Foundation for Statistical Computing, Vienna, Austria, https://www.R-project.org/). Trends in RSV for diseases were illustrated using line graphs. For treatment modalities, RSV data were grouped into two time periods: January 2014-December 2018 and January 2019-December 2023. The normality of the variables was assessed using the Shapiro-Wilk test. Comparisons between the two periods were performed using the Mann-Whitney U test, with p < 0.05 considered statistically significant.

## Results

**Figure 1:**
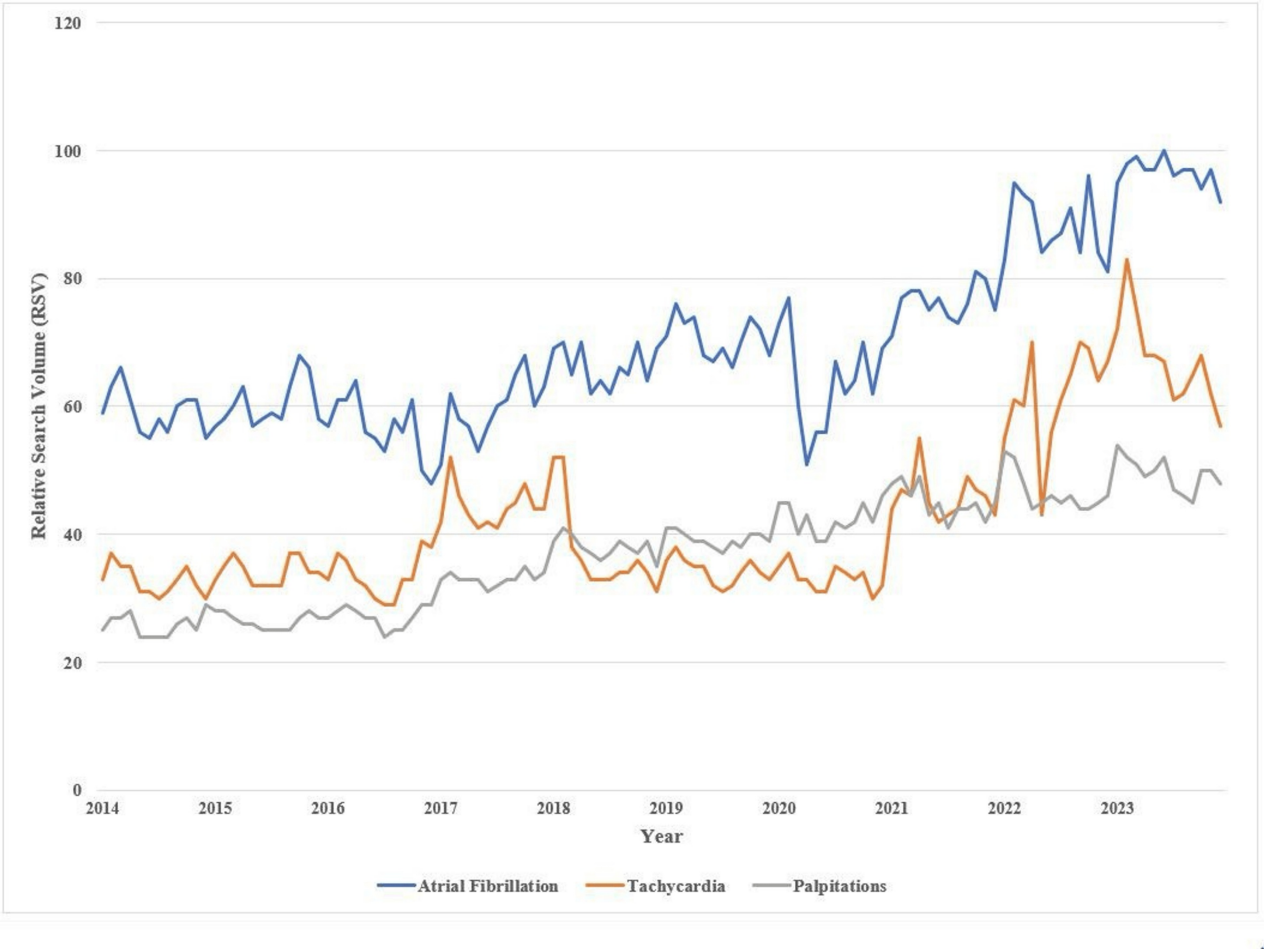
Changes in the relative search volume (RSV) of cardiology diseases by years in the United States using Google Trends data

Figure 1 illustrates the annual changes in the RSV of cardiology-related diseases in the United States, based on Google Trends data.

The RSV for all disease-related terms showed an overall increasing trend from 2014 to 2023. The RSV for "AF" remained relatively stable from 2014 to 2018, followed by a gradual increase until 2023. The RSV for "tachycardia" also remained relatively constant from 2014 to 2021, except for a sudden rise in the 2017-2018 period, and then increased rapidly from 2021 to 2023. In contrast, the RSV for "palpitations" demonstrated a steady, moderate increase throughout the study period. Among the three terms, "AF" consistently had the highest RSV compared to "tachycardia" and "palpitations."

Table 1 presents the yearly distribution of search frequency for various medications, procedures, and surgical interventions related to AF.

**Table 1:** Distribution of search frequency for medications and procedures/surgeries for atrial fibrillation by years p < 0.05 is considered statistically significant.

Terms	January 2014-December 2018 (median (IQR))	January 2019- December 2023 (median (IQR))	p-value (Mann-Whitney U test)
Medications			
Digoxin	45.00 (42.00-49.75)	48.00 (42.25-52.75)	0.109
Metoprolol	55.00 (51.00-69.00)	82.00 (77.00-88.00)	<0.000
Verapamil	76.00 (67.25-79.00)	73.50 (69.00-79.00)	0.929
Diltiazem	62.00 (58.00-67.75)	79.00 (74.00-85.00)	<0.000
Procainamide	72.00 (65.00-77.75)	72.00 (64.25-81.75)	0.937
Disopyramide	51.00 (43.00-67.75)	50.00 (47.00-54.75)	0.560
Flecainide	47.00 (40.00-58.25)	74.00 (69.00-87.00)	<0.000
Propafenone	86.00 (81.00-91.00)	80.00 (74.00-84.00)	<0.000
Sotalol	72.50 (65.25-81.50)	88.00 (81.25-92.00)	<0.000
Amiodarone	59.50 (54.00-62.75)	75.00 (68.00-83.75)	<0.000
Dofetilide	70.00 (65.00-77.00)	83.00 (72.00-89.75)	<0.000
Aspirin	68.50 (63.25-73.00)	83.00 (79.00-87.75)	<0.000
Warfarin	85.00 (80.00-92.00)	68.00 (64.00-73.75)	<0.000
Procedure and surgery			
Cardioversion	53.50 (49.25-62.00)	74.00 (67.00-81.75)	<0.000
Pulmonary vein ablation	39.50 (0.00-53.00)	41.00 (32.00-51.00)	0.337
Artificial cardiac pacemaker	58.00 (53.25-64.00)	76.00 (69.00-84.75)	<0.000
Left atrial appendage occlusion	28.00 (19.00-40.75)	72.00 (62.25-80.00)	<0.000
Maze procedure	71.50 (64.25-82.00)	70.00 (65.00-75.75)	0.198

With regard to medications, the RSV for "metoprolol," "diltiazem," "flecainide," "sotalol," "amiodarone," "dofetilide," and "aspirin" has significantly increased, while the RSV for "propafenone" and "warfarin" has shown a significant decline over the past five years (January 2019-December 2023) compared to the previous five years (January 2014-December 2018).

Among the procedures, "cardioversion," "artificial cardiac pacemaker," and "left atrial appendage occlusion" demonstrated a significant increase in RSV over the past five years (January 2019-December 2023) compared to the previous five years (January 2014-December 2018).

## Discussion

A cross-sectional study was conducted in July 2024 to assess public interest in AF and its treatment modalities in the United States. Google Trends was utilized as a data source to track public interest over time. The findings, based on comparisons between two five-year periods (i.e., 2014-2018 and 2019-2023), suggest significant differences in the RSV for medications and procedures (except for digoxin, verapamil, procainamide, disopyramide, pulmonary vein ablation, and maze procedure).

Health-related web content is widely accessible and often perceived by users as more convenient than waiting for a physician consultation. The most searched health term globally is edema, followed by chest pain. In the United States, chest pain is the second most common presenting symptom in emergency departments, accounting for 5.2% of visits. Women are more active than men in seeking health-related information online [8]. The Internet has increasingly become a primary health resource for both patients and healthcare professionals. Google Trends has emerged as a valuable tool for surveillance and analysis of public interest in health topics, including diseases, epidemics, and outbreaks. Its open-access nature and user-friendly interface have made it widely adopted among researchers studying behavioral trends [9]. Google Trends enables the identification of which diseases and treatment modalities generate the most public interest and search activity.

AF is recognized as the most common cardiac arrhythmia and is a significant cause of death, primarily due to stroke and embolism. According to a study, its prevalence can be estimated at up to 33.5 million. A significant pathophysiological component related to AF is atrial fibrosis, which contributes to disease recurrence, resistance to treatments, and complications [10]. Although antiarrhythmic drugs have long been central to rhythm control in AF management, the use of catheter ablation has been increasing in recent years. Between 2004 and 2016, the use of antiarrhythmic medications tripled, with over 63 million individuals receiving them during this period [11]. Pharmacological management often requires time to achieve therapeutic effect, and outcomes may be variable, particularly when the underlying pathology is not fully understood. In recent times, surgical interventions have gained prominence in the management of AF. One notable example is the Cox-Maze procedure, which has undergone significant refinement and continues to evolve as a rhythm control strategy [12]. Patients with AF should be screened for thromboembolic risk, regardless of the chosen symptom management approach. Anticoagulation remains a cornerstone of stroke prevention in AF. In recent years, novel oral anticoagulants (NOACs), including dabigatran and the factor Xa inhibitors rivaroxaban, apixaban, and edoxaban, have gained favor over warfarin due to their rapid onset of action, predictable pharmacokinetics, and reduced need for monitoring [13,14].

A 2020 study investigated geographic and temporal trends in YouTube and Google Search activities related to “psoriasis” and “atopic eczema.” The findings revealed that since 2008, YouTube search activity for “psoriasis” had tripled, while searches for “atopic eczema” declined. In contrast, Google Trends data showed an increase in search activities for “psoriasis” and “atopic eczema” [15]. A similar study done in 2021 examined Google Search trends related to COVID-19 and clotting risk, reporting a statistically significant Spearman correlation between search volume and increased clinical focus on thromboprophylaxis and anticoagulation therapy for COVID-19 patients [16]. Social media platforms have also played a growing role in healthcare communication. Twitter, in particular, has become widely used for medical discourse. A 2023 study highlighted the platform’s benefits in cardiology, noting its ability to facilitate virtual engagement between healthcare professionals and patients globally [17].

Limitations

There are several limitations to using Google Trends for health-related analysis. The tool presents data in terms of RSV rather than absolute search counts, which may limit the precision of interpretation. Additionally, some individuals have no access to the Internet, a computer, or any smart devices. Most importantly, we cannot access the quality and authenticity of the data available on websites, and the treatment modalities searched on Google can be common to many different diseases. While increasing RSV reflects growing public interest, it may also be shaped by external influences such as media reports, clinical guideline updates, or marketing efforts. These trends, though not direct measures of prevalence or clinical use, can help healthcare professionals anticipate patient concerns and develop targeted education strategies that align with the public’s evolving information needs.

## Conclusions

The data demonstrates a consistent increase in the RSV for cardiovascular-related terms between 2014 and 2023, with "AF" consistently emerging as the most searched term. Over the past five years, there has been a notable rise in the RSV for medications like "metoprolol," "diltiazem," "flecainide," "sotalol," "amiodarone," "dofetilide," and "aspirin," while searches for "propafenone" and "warfarin" have declined. Additionally, there has been a significant growth in the RSV for procedures such as "cardioversion," "artificial cardiac pacemaker," and "left atrial appendage occlusion" during this time. Considering that Google Trends data offers valuable insights into public interest in various treatment options, public health authorities need to incorporate this data into their strategic planning. This approach would allow them to create and distribute accurate and relevant guidelines online, ensuring that the public has access to trustworthy information. Such measures are important as these trends can impact treatment decisions and shape patient expectations, highlighting the need for proactive communication from public health officials. Moreover, health insurance providers should take these trends into account when updating coverage policies, ensuring they reflect evolving medical practices and patient needs, ultimately promoting informed and effective healthcare decisions.

Future research could build on these findings by correlating search interest with real-world clinical data, evaluating the influence of media or health campaigns, and expanding keyword selection to include newer therapies. Additionally, exploring public interest across multiple platforms and using time-series analyses could offer deeper insights into evolving health information-seeking behaviors.
